# Advancing microbiota therapeutics: the role of synthetic biology in engineering microbial communities for precision medicine

**DOI:** 10.3389/fbioe.2024.1511149

**Published:** 2024-12-04

**Authors:** Asiya Nazir, Fathima Hasnain Nadeem Hussain, Afsheen Raza

**Affiliations:** Department of Biomedical Sciences, College of Health Sciences, Abu Dhabi University, Abu Dhabi, United Arab Emirates

**Keywords:** synthetic biology, gut microbiota therapeutics, CRISPR/Cas9 gene editing, metabolic engineering, personalized medicine

## Abstract

Over recent years, studies on microbiota research and synthetic biology have explored novel approaches microbial manipulation for therapeutic purposes. However, fragmented information is available on this aspect with key insights scattered across various disciplines such as molecular biology, genetics, bioengineering, and medicine. This review aims to the transformative potential of synthetic biology in advancing microbiome research and therapies, with significant implications for healthcare, agriculture, and environmental sustainability. By merging computer science, engineering, and biology, synthetic biology allows for precise design and modification of biological systems via cutting edge technologies like CRISPR/Cas9 gene editing, metabolic engineering, and synthetic oligonucleotide synthesis, thus paving the way for targeted treatments such as personalized probiotics and engineered microorganisms. The review will also highlight the vital role of gut microbiota in disorders caused by its dysbiosis and suggesting microbiota-based therapies and innovations such as biosensors for real-time gut health monitoring, non-invasive diagnostic tools, and automated bio foundries for better outcomes. Moreover, challenges including genetic stability, environmental safety, and robust regulatory frameworks will be discussed to understand the importance of ongoing research to ensure safe and effective microbiome interventions.

## 1 Introduction

Synthetic biology is a multidisciplinary field that integrates principles from computer science, engineering, biology, and other domains to design and manipulate biological systems (Mukherji and Van, 2009; Ruder et al., 2011; Ezzamouri et al., 2021). By applying engineering concepts, synthetic biology facilitates the creation of novel artificial biological systems or the redesign of existing ones to perform specific functions. This approach leverages advanced techniques such as genome editing, particularly CRISPR/Cas9, and computational modeling (Garner, 2021; Singh et al., 2019; Gardner, 2013). In healthcare, it enables personalized therapies (Kumar et al., 2018; Yadav and Chauhan, 2022), development of novel treatments, and enhanced diagnostic tools (Yan et al., 2023; Yadav and Chauhan, 2022). In agriculture, it offers solutions for increasing crop yields (Marik et al., 2024), enhancing stress resilience, and improving nutrient utilization (Sedeek et al., 2019). Industrial applications encompass sustainable production of materials, biofuels, and biochemicals (Shi et al., 2022), while environmental applications include pollution remediation and biosensor development (Chen and Silver, 2012; Malaviya et al., 2023).

Synthetic biology offers numerous benefits, but it also encounters technological, ethical, and regulatory obstacles. Ethical concerns revolve around the creation of synthetic organisms and the potential for unintended genetic changes, emphasizing the need for strong regulatory frameworks to ensure safe application (National Academies of Sciences, Engineering, and Medicine, 2018). Synthetic biology in gut microbiome engineering, for example, necessitates a thorough examination of biosafety standards and ethical frameworks to avoid unintended ecological or health consequences (Ou and Guo, 2023). While, technological challenges include improving the predictability and reliability of engineered systems and scaling them for industrial applications (Wang et al., 2013). Synthetic biology can also be harnessed to engineer probiotics that target infectious agents, produce therapeutic compounds (Chauhan, 2023), or modify gut microbiota (Yadav et al., 2023) to improve outcomes in diseases such as inflammatory bowel disease and metabolic disorders (Mimee et al., 2018; Zhou et al., 2020). As such, microbial communities, engineered through synthetic biology, may replicate natural ecosystems, facilitating the study of microbial interactions and host-microbiota dynamics (Mejía-Caballero et al., 2021). Thus, the intersection of synthetic biology and microbiome research presents significant opportunities for advancing biotechnology (Kumar and Chauhan, 2021; Yadav and Chauhan, 2022), environmental sustainability, and healthcare. Furthermore, modified microbes can serve as biosensors for ecological monitoring and diagnostic purposes (Paczesny et al., 2020; Sharma et al., 2023).

The human microbiota, primarily composed of bacteria residing in the gut, skin, and mucosal surfaces (Yadav M. and Chauhan N. S., 2024), play a crucial role in digestion, metabolism, immune regulation, and neurological functions (Belkaid and Segre, 2014; Kumar and Chauhan, 2022). Dysbiosis, characterized by an imbalance in microbiota composition, has been linked to various diseases, including neurodevelopmental disorders, diabetes, inflammatory bowel disease, and obesity (Hou et al., 2022; Yadav P. and Chauhan N. S., 2024). Understanding the interactions between microbiota and host is essential for the development of microbiome-based therapeutics (Yadav and Chauhan, 2022), such as prebiotics and probiotics, aimed at restoring microbial balance and enhancing health (Hemarajata and Versalovic, 2013; Zhou et al., 2024; Hitch et al., 2022). Major challenges and prospects highlighting the necessity for continued technological, ethical, and regulatory hurdles while advancing microbiota-based therapeutics are also discussed for better insight on the microbiome.

## 2 Synthetic biology tools

Synthetic biology combines science and engineering principles to develop novel biological systems, utilizing advanced technologies such as CRISPR, TALENs and ZFNs as summarized in Table 1 (Jeong et al., 2023). This multidisciplinary approach enables precise manipulation of organisms, facilitating innovative applications across medicine, agriculture, and environmental science. As the field progresses, it holds significant potential to address critical global challenges. For instance, in healthcare, synthetic biology has enabled the rapid development of mRNA vaccines, as seen during the COVID-19 pandemic, which highlights its potential for combating emerging infectious diseases (Pfeifer et al., 2023). Additionally, engineered organisms are being used to produce new antimicrobial agents to counter antibiotic-resistant pathogens, a growing global threat (Dana et al., 2016).

**TABLE 1:** Comparison of CRISPR/Cas9, TALENs, and ZFNs in genome editing technologies.

	CRISPR/Cas9	TALENs (transcription activator-like effector nucleases)	ZFNs (zinc finger nucleases)
Mechanism of action	Cas9 nuclease is guided to DNA loci by guide RNA	DNA is bound by TALEs, and double-strand breaks are caused by the FokI nuclease	DNA is bound by zinc finger domains, and FokI nuclease cleaves
DNA Repair Pathways	DSBs repaired using NHEJ or HDR	DSBs repaired using NHEJ or HDR	DSBs modified using NHEJ or HDR
DNA targeting strategy	RNA-based	TALE-derived DNA-binding domains	Zinc finger motifs customized for specific sequences
Applications	Gene insertion, modification, multiplexing and gene knockout	Genome editing in differenet cell types and different organisms	Precision editing
Uses	Trials involving humans, animals, plants and various therapeutic strategies	Genetic engineering	Genetic research, early genetic studies and therapeutic applications

### 2.1 Gene editing tools

Gene editing techniques have ushered in a new era of precision biology, providing exceptional capabilities for the effective and accurate modification of genetic sequences across a range of organisms, from microbes to animals and plants (Chauhan et al., 2020). These tools empower researchers to modify individual genes, introduce new genetic sequences, and repressed genes with unprecedented efficiency and precision (Gaj et al., 2016; Ahmed et al., 2018). The advent of gene editing technologies has revolutionized biological research, enabling investigation of gene function, disease modelling, and potential discovery of novel therapeutics for rare and common genetic disorders (Li et al., 2020).

CRISPR/Cas9 technology, which was discovered in 2012, revolutionized genome editing by using a bacterial immune system to make precise genetic changes. This genetic manipulation tool is derived from the adaptive immune system of bacteria like *Streptococcus pyogenes* and *Escherichia coli* that helps defend it against viral infections and plasmids (Marraffini and Sontheimer, 2008; Doudna and Charpentier, 2014; Rodríguez-Rodríguez et al., 2019). Flexible design has enabled widespread application across various experimental models, including plants, laboratory animals, cell lines, and even human clinical trials (Li et al., 2020). A guide RNA directs the Cas9 nuclease to particular DNA sites, causing double-strand breaks (DSBs) that may be repaired by HDR or NHEJ (Rodríguez-Rodríguez et al., 2019; Chauhan, 2022; Walter and Engelke, 2002). Because of its simplicity and ability to multiplex, this approach has been widely used in a variety of experimental models, including human clinical trials (Li et al., 2020). TALENs (Transcription Activator-Like Effector Nucleases) are another type of customizable nucleas that combine DNA-binding regions from transcription activator-like effectors with a FokI nuclease, providing greater specificity and lower toxicity than zinc finger nucleases (ZFNs) (Christian et al., 2010; Gaj et al., 2013). TALENs (Transcription Activator-Like TALENs) also cause DSBs, which allow for precise genome alterations in a variety of species. ZFNs, one of the first designed nucleases, are made up of FokI and zinc finger DNA-binding domains. They enable targeted gene editing by inducing DSBs, however their design is more labor-intensive than CRISPR/Cas9 and TALENs because zinc finger domains must be customized for each target sequence, limiting their utility regardless of their precision (Koijam et al., 2024; Paschon et al., 2019).

### 2.2 DNA assembly and synthesis

**FIGURE 1:**
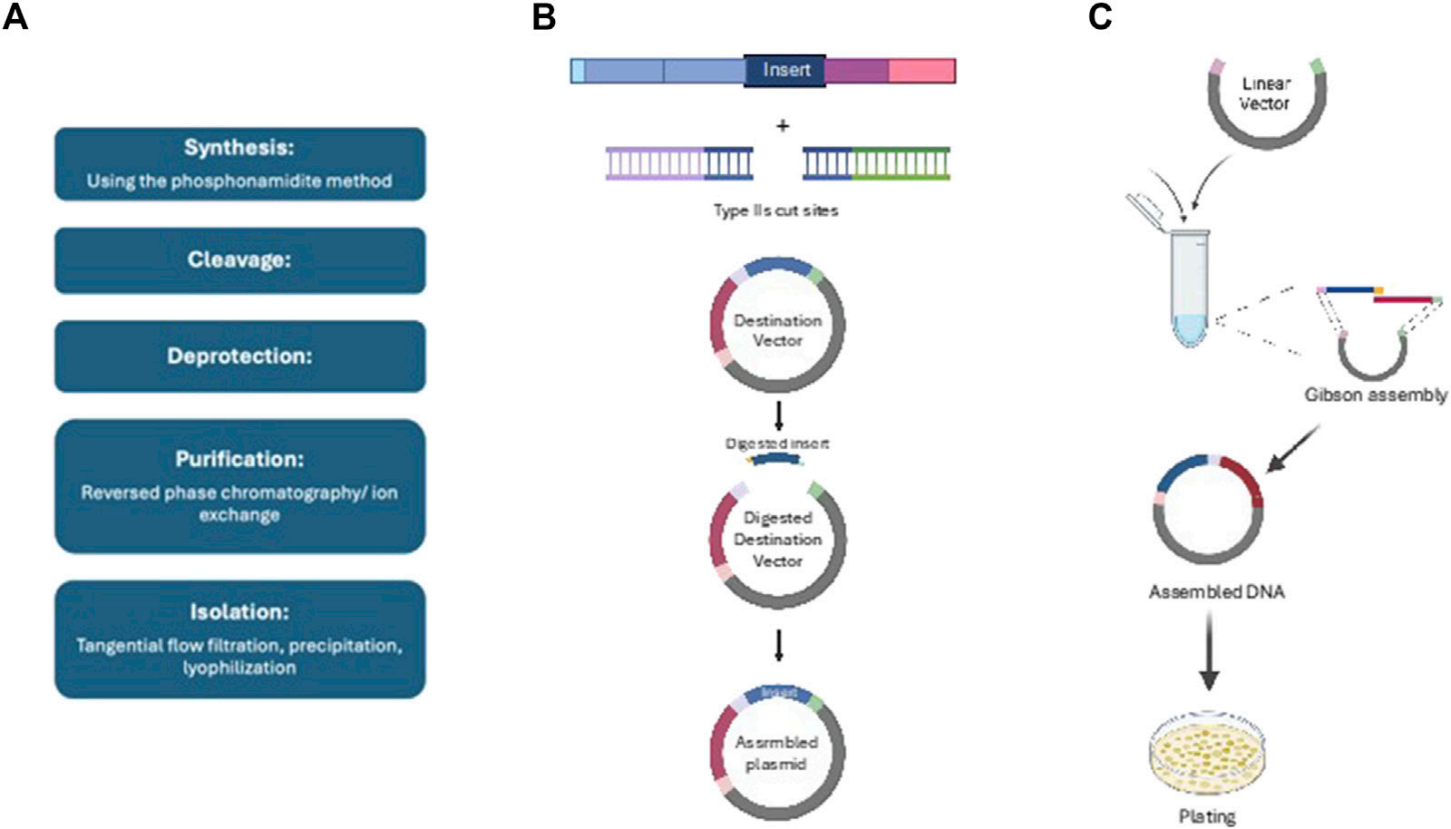
Summary of different DNA assemblies and synthesis: **(A)** Gibson assembly, **(B)** Golden gate assembly, **(C)** Synthetic oligonucleotide synthesis.

DNA assembly, which is required for the production of DNA molecules, happens both spontaneously and in the laboratory (Robinson et al., 2018). In natural systems, initiator proteins attach to replication origins, unwinding DNA and enabling polymerases to precisely synthesis new strands with the help of different enzymes (Valenzuela-Amaro et al., 2023). In the lab, synthetic biology approaches such as Gibson and Golden Gate Assembly employ particular enzymes to effectively put together DNA fragments, typically with sequencing and quality checks to verify correctness, shown in Figure 1 (Alberts, 2002; Chao et al., 2015). DNA synthesis is classified into two types: biological, which employs enzymes to duplicate DNA with high fidelity, and synthetic, which uses phosphonamidite chemistry to produce bespoke, high-throughput DNA (Nair and Gonzalez-Angulo, 2015). Synthetic DNA has many uses in science, medicine and biotechnology involving CRISPR development, recombinant proteins, genetic diagnostics and mRNA vaccines (Yadav and Chauhan, 2021; Lee, 2023).

Gibson Assembly is a DNA assembly technology that effectively combines numerous DNA fragments in a single reaction using enzymatic techniques. It uses overlapping sequences processed by an exonuclease to generate 5′overhangs, which facilitates annealing, gap filling by DNA polymerase, and sealing by DNA ligase, resulting in smooth constructions (Semkum et al., 2023; Gibson et al., 2009). This approach is very useful for gene insertion and genome synthesis because to its accuracy, albeit careful fragment design and verification by sequencing are required (Shao and Zhao, 2012; Thomas et al., 2015; Olszakier and Berlin, 2022). Golden Gate Assembly, on the other hand, employs Type IIS restriction enzymes to generate specialized sticky ends that enable the smooth assembly of many fragments in a single process. It has a high assembly efficiency rate, frequently exceeding 90%, and is used for gene insertion and pathway creation (Bird et al., 2022). The approach necessitates careful creation of DNA fragments containing recognition sites, as well as rigorous quality control by sequencing (Marillonnet and Grützner, 2020). Finally, synthetic oligonucleotide synthesis creates bespoke DNA or RNA sequences via solid-phase chemical synthesis, which involves adding nucleotides to a solid support. This technique is critical for applications such as PCR primers and antisense agents because it allows for quick and precise sequence production, while verification methods such as sequencing and mass spectrometry ensure product integrity (Chauhan et al., 2022; Hughes and Ellington, 2017; Tang et al., 2013; Hoose et al., 2023; Masaki et al., 2022; Ma et al., 2012).

### 2.3 Promoters and regulatory elements

Promoters are specific DNA regions at the beginning of genes that allow RNA polymerase and transcription factor binding, so commencing transcription (Minchin and Busby, 2013). The TATA box and initiator (Inr) element are key factors within promoters, whereas upstream elements like as the GC and CAAT boxes govern transcription rates by interacting with regulatory proteins (Villao-Uzho et al., 2023; Dao and Spicuglia, 2018). Enhancers are a type of regulatory element that increases transcriptional activity by looping DNA, allowing them to interact with promoters even when they are thousands of base pairs distant (Pennacchio et al., 2013). Insulators block enhancers from activating adjacent genes, preserving transcriptional specificity. LCRs help to organize the expression of gene clusters within certain chromosomal areas (Maurya, 2021; Mercatelli et al., 2020). Advanced strategies like Hi-C have demonstrated that genome spatial organization is critical in promoter-enhancer interactions, stressing the three-dimensional chromatin structure’s influence on gene regulation (Belton et al., 2012). Furthermore, epigenetic modifications such as DNA methylation and histone modifications change gene accessibility and transcriptional activity without changing the DNA sequence, resulting in an intricate regulatory system that governs the expression of genes in response to cellular and environmental cues as demonstrated in Table 2 (Panigrahi and O'Malley, 2021).

**TABLE 2:** Overview of synthetic promoters, inducible/repressible enzymes, and RNA-Based regulatory elements (riboswitches and ribozymes).

	Synthetic promoters	Inducible and repressible enzymes	Riboswitches and ribozymes
Definition	Engineered promoters that combine cis-regulatory components	Enzymes regulated by environmental factors (inducers or corepressors)	RNA elements that control gene expression and carry out enzymatic reactions
Components	Enhancers, silencers, operator and core promotor	Inducible: promoter/operator controlled by inducers Repressible: repressor/corepressor prevents transcription	Riboswitches RNA segments that bind small molecules to regulate the structure of mRNA Ribozymes RNA molecules that catalyze splicing and cleavage
Mechanism	Modular design enables precise responses to stimuli	Inducible: inducer binds the repressor, relieving inhibition Repressible: Corepressor activates repressor to prevent transcription	Riboswitches: Modify mRNA structure to regulate translation or transcription Ribozymes catalyze RNA cleavage, splicing, and modification
Applications	Crop breeding, metabolic engineering, molecular pharmacology, and synthetic biology	Biotechnology (e.g., recombinant protein production), pharmaceuticals, biosensors	RNA-based treatments, environmental monitoring, medical diagnostics, and biosensors

Synthetic promoters, inducible and repressible enzymes, riboswitches, and ribozymes are effective tools for precisely regulating genes in molecular biology and biotechnology. Synthetic promoters are DNA elements constructed to influence transcription with high efficiency by integrating several cis-regulatory elements, allowing for targeted gene expression in response to environmental stimuli like as temperature and chemicals. They serve important roles in molecular pharmacology, crop breeding, and metabolic engineering. Inducible enzymes are controlled by inducers that remove repressor proteins from operator areas, permitting transcription; they are widely employed in recombinant DNA technology for regulated protein synthesis and cost-effective manufacture (Robinson, 2015). Repressible enzymes, on the other hand, are regulated by corepressors that attach to repressors and lower synthesis when end products are adequate, which aids in sectors such as antibiotic manufacture and agricultural trait management. Riboswitches, which are found in mRNA 5′UTRs, bind metabolites and influence gene expression by conformational changes. They have potential in biosensing and synthetic gene regulation (Kavita and Breaker, 2023; Yu et al., 2024). Ribozymes, RNA molecules with enzymatic activities, have critical roles in RNA processing, gene therapy, and RNA manipulation, with applications in therapeutic design and genetic engineering (Vlasova-St. Louis, 2023). Together, these components increase gene control and allow for novel applications in diagnostics, medicine, and biotechnology.

### 2.4 Metabolic engineering tools

Metabolic engineering tools are essential for the optimization and construction of metabolic pathways aimed at producing valuable compounds, including pharmaceuticals, biofuels, and specialty chemicals (Chae et al., 2017). These advancements enable the development of microbial cell factories capable of efficiently synthesizing a diverse array of chemicals and materials, such as biofuels, bulk and fine chemicals, polymers, amino acids, natural products, and therapeutic agents. Systems metabolic engineering integrates traditional metabolic engineering with principles from synthetic biology, systems biology, and evolutionary engineering, creating a comprehensive approach to enhance metabolic function and compound production (Ko et al., 2020).

Pathway engineering focuses on improving and creating metabolic pathways to increase the synthesis of valuable chemicals by changing genes to shift metabolic fluxes (Fisher et al., 2014). Enzyme engineering improves enzyme activity and stability (Malaviya et al., 2023), whereas pathway optimization uses metabolic flow analysis to increase desired metabolite outputs while minimizing byproducts (Iwatani et al., 2008; Eriksen et al., 2014). Computational modeling helps to simulate the consequences of genetic alterations on pathways, allowing for the creation of more efficient biofuel and medicinal processes (Alper and Avalos, 2018). Flux Balance Analysis (FBA) examines and improves metabolic networks by modeling metabolite flow and optimizing or decreasing certain metabolic fluxes under restrictions, hence improving metabolic route design (Rajvanshi and Venkatesh, 2013; Orth et al., 2010; Sahu et al., 2021). Pathway engineering focuses on improving and creating metabolic pathways to increase the synthesis of valuable chemicals by changing genes to shift metabolic fluxes (Fisher et al., 2014). Enzyme engineering improves enzyme activity and stability (Malaviya et al., 2023), whereas pathway optimization uses metabolic flow analysis to increase desired metabolite outputs while minimizing byproducts (Iwatani et al., 2008; Eriksen et al., 2014). Computational modeling helps to simulate the consequences of genetic alterations on pathways, allowing for the creation of more efficient biofuel and medicinal processes (Alper and Avalos, 2018). Flux Balance Analysis (FBA) examines and improves metabolic networks by modeling metabolite flow and optimizing or decreasing certain metabolic fluxes under restrictions, hence improving metabolic route design (Rajvanshi and Venkatesh, 2013; Orth et al., 2010; Sahu et al., 2021).

### 2.5 Synthetic biology platform

Synthetic biology platforms are specialized infrastructures that facilitate the development, integration, and application of synthetic biology techniques, allowing for the creation of new biological systems and organisms in fields such as agriculture, healthcare, and environmental sciences (MacDonald and Deans, 2016). CRISPR/Cas9, synthetic gene circuits, and modular DNA are among the techniques available for gene editing, genome assembly, and pathway design on these systems. High-throughput technologies enable quick screening, DNA synthesis, protein expression, and metabolic engineering, while sophisticated computer models help anticipate and optimize gene interactions and metabolic pathways (Bak et al., 2022; Kwon et al., 2024). These platforms, equipped with automation and robotics, facilitate both large-scale manufacturing and small-scale research. They serve as collaborative hubs that stimulate multidisciplinary innovation and connect researchers with industry (Brooks and Alper, 2021; Si and Zhao, 2016).

Biofoundries, design tools, and standardized biological pathways are transforming synthetic biology by improving automation, computer modeling, and modular genetic engineering. Biofoundries improve synthetic biology through automated processes, robotic systems, and computational tools, allowing for high-throughput screening and precision biological product creation (Lee et al., 2023; Tellechea-Luzardo et al., 2022). Gene editing, cloning, and sequence analysis are supported by design tools such as Benchling and Geneious, allowing for more efficient and collaborative research (Bosley et al., 2021; Kearse et al., 2012). BioBricks are standardized genetic modules that allow for the smooth integration of genetic components, which is critical for complex genetic circuits utilized in a variety of applications such as metabolic engineering and instructional aids (Yamazaki et al., 2017; Anderson et al., 2010; Radde et al., 2024). These discoveries work together to expedite synthetic biology, allowing for fast prototyping and modular assembly of genomic structures. In microbiota therapies, synthetic biology allows for the engineering of microbial communities, resulting in tailored medicines for ailments such as obesity and inflammatory disorders, with tools such as BioBricks facilitating fast microbial therapy development (Røkke et al., 2014; Singh J. et al., 2024; Chauhan, 2023).

## 3 Importance of gut microbiota

**FIGURE 2:**
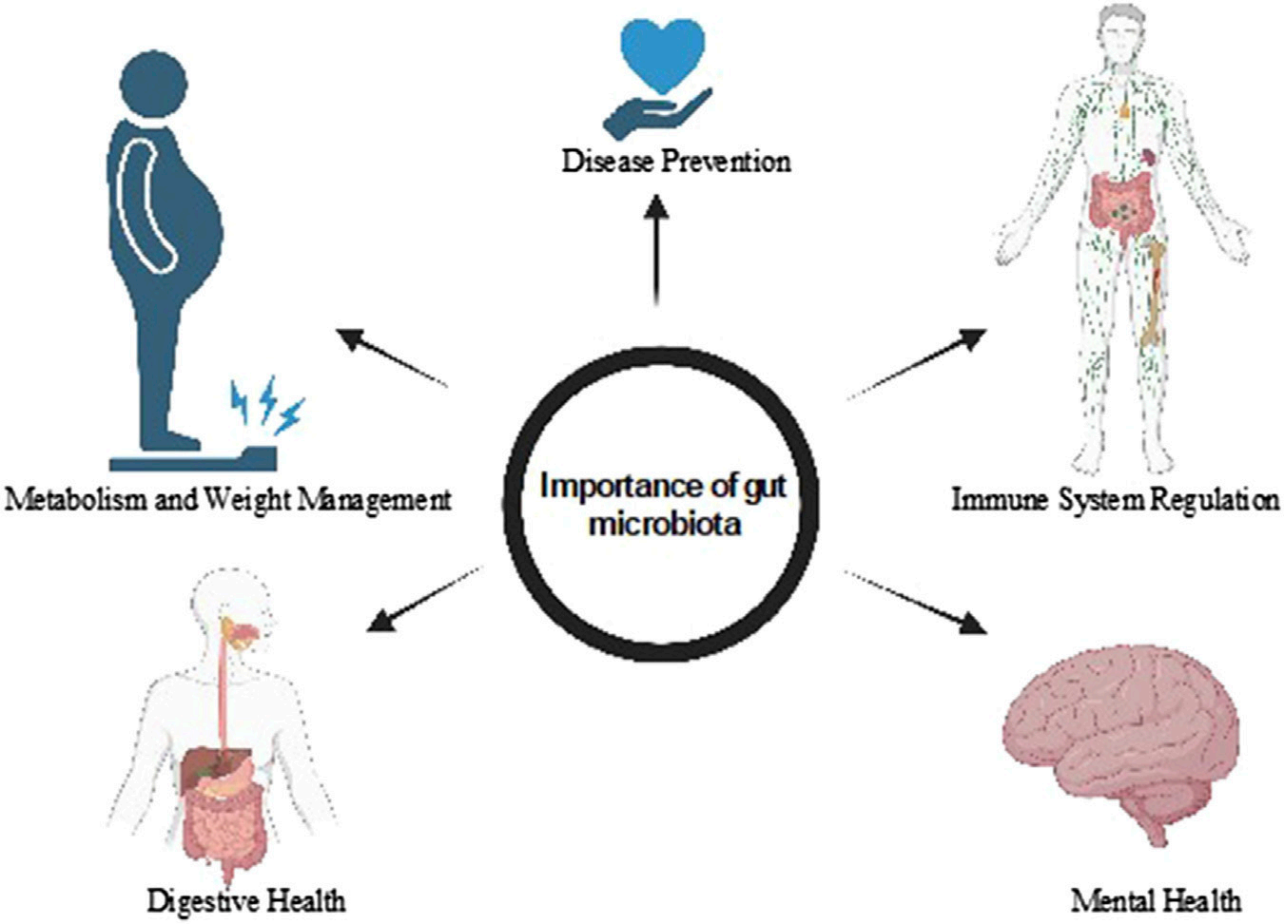
Role of gut microbiota in digestion and food absorption, immune system modulation, mental health, and the creation of beneficial metabolites.

The human gut harbors a vast and diverse community of microbes (Kumar et al., 2015), collectively forming a complex microbial ecosystem that plays a pivotal role in human health, summarized in Figure 2. This gut microbiota is increasingly recognized as a vital organ in its own right, functioning as a multidirectional axis linking the gut to other organ systems throughout the body (Yadav et al., 2022a). The predominant phyla within the typical human gut microbiota are Bacteroidetes and Firmicutes, which together account for a significant proportion of the microbial community (Afzaal et al., 2022). In early life, an infant’s gut microbiota appears somewhat arbitrary, influenced by factors such as mode of delivery, diet, and environmental exposures. However, by around 3 years of age, this microbial community begins to stabilize and more closely resemble that of an adult (Jandhyala, 2015). This maturation process is crucial, as the composition and diversity of gut microbiota directly influences host health and disease susceptibility (Rooks and Garrett, 2006; Madhu et al., 2023). The gut microbiota axis regulates intricate host-microbe interactions and communicates with various physiological systems, including neuronal, endocrine, immunological, humoral, and metabolic pathways (Wang et al., 2017; Chauhan et al., 2018). For instance, gut microbes can produce metabolites like short-chain fatty acids (SCFAs), which not only provide energy to colonocytes but also modulate immune responses and influence brain function through the gut-brain axis. Furthermore, alterations in gut microbiota composition have been linked to various health conditions, including obesity, diabetes, inflammatory bowel disease (Singh A. et al., 2024), and mental health disorders, underscoring the importance of this microbial ecosystem in maintaining overall health and homeostasis (Kumar et al., 2020). As research continues to uncover the complexities of gut microbiota interactions, it opens new avenues for therapeutic interventions to restore microbial balance and improve health outcomes.

### 3.1 Immune system regulation

The gut microbiota plays a crucial role in efficient functioning of the immune system as approximately 70% of the body’s immune cells reside within the gut. This unique arrangement allows the microbiota to interact closely with immune cells, training and regulating immune responses to maintain homeostasis and protect the host from pathogens (Wiertsema et al., 2021). Through various mechanisms, the gut microbiota influences immune cell development and the production of immune-signaling molecules, including cytokines and chemokines. These interactions are essential for shaping innate and adaptive immunity, ultimately affecting the body’s capacity to combat infections and prevent the onset of autoimmune diseases. For instance, certain beneficial microbes can stimulate the production of regulatory T cells, which help maintain immune tolerance and prevent excessive inflammatory responses. A healthy and diverse microbiome promotes a balanced immune system, enhancing the body’s ability to fend off infections and reducing the risk of chronic diseases. Dysbiosis through disruptions in the composition or function of the gut microbiota has been linked to various health conditions, including allergies, inflammatory bowel diseases, and even metabolic disorders (Maciel-Fiuza et al., 2023). Furthermore, short-chain fatty acids (SCFAs), synthesized by the gut microbiota contributes immune modulation leading to an efficient response against foreign agents. These metabolites enhance the integrity of the gut barrier, reducing systemic inflammation and supporting overall immune health. As research continues to elucidate the complex interplay between gut microbiota and the immune system, it becomes increasingly clear that maintaining a healthy microbiome is vital for fostering robust immune function and promoting long-term health. This understanding opens new avenues for therapeutic interventions aimed at restoring microbial balance and enhancing immune resilience.

### 3.2 Disease prevention

A diverse and balanced gut microbiota is critical in protecting the host from harmful organisms, promoting overall health. The human gut is home to many bacteria, primarily non-pathogenic species, which engage in symbiotic interactions with the host. These interactions are essential for modulating the immune system enhancing the host’s defense mechanisms against pathogenic invasion (Afzaal et al., 2022). The gut microbiota acts as a dynamic barrier, preventing the colonization of harmful pathogens through various mechanisms (Yadav et al., 2020b). For example, beneficial bacteria can outcompete pathogenic bacteria for nutrients and binding sites, produce antimicrobial substances that inhibit pathogenic agents, and activate the immune system to respond effectively. This protective function underscores the importance of maintaining a healthy microbial ecosystem within the gut. However, disruptions in microbiota diversity—often caused by factors such as poor dietary choices, antibiotic use (Ahmed et al., 2013; Ahmed et al., 2014), and environmental influences—can significantly impair these protective mechanisms. As such, dysbiosis has been associated with an increased susceptibility to a wide range of health issues, including chronic inflammatory conditions, cardiovascular diseases, and even certain types of cancer (Hou et al., 2022). For instance, an imbalanced gut microbiota may lead to an overgrowth of pathogenic bacteria, resulting in chronic inflammation, which is a known risk factor for conditions such as inflammatory bowel disease (IBD) and metabolic syndrome. Additionally, the loss of microbial diversity can hinder the production of beneficial metabolites like short-chain fatty acids (SCFAs), which have anti-inflammatory properties and are vital for maintaining gut health. Research has also indicated that dysbiosis can influence the progression of diseases like obesity and diabetes by affecting metabolic pathways and the body’s inflammatory responses. Furthermore, emerging studies suggest a potential link between gut microbiota composition and the risk of certain malignancies, highlighting the intricate connections between microbial health and systemic disease outcomes (Rasgania et al., 2024).

### 3.3 Digestive health

The gut microbiota is essential for digesting complex carbohydrates and dietary fibers via microbial fermentation that transforms these indigestible substrates into short-chain fatty acids (SCFAs) such as acetate, propionate, and butyrate. These fatty acids are crucial for maintaining gut health, regulating energy metabolism, and modulating inflammation (den Besten et al., 2013). SCFAs are also vital for host energy metabolism and as an energy source for colonic epithelial cells that facilitates overall metabolic homeostasis. By promoting the absorption of nutrients and influencing lipid metabolism, SCFAs help maintain a balanced energy state within the body. Additionally, these fatty acids play a pivotal role in regulating the immune response, acting as signaling molecules that help modulate inflammation and protect against inflammatory disorders. A healthy gut microbiome can significantly reduce the risk of various digestive disorders, including inflammatory bowel disease (IBD) and irritable bowel syndrome (IBS) by preventing dysbiosis and subsequent exacerbation of these conditions (Rowland et al., 2018; Singh J. et al., 2024). For instance, in IBD, an altered microbial composition can lead to an inappropriate immune response to gut microbiota, resulting in chronic inflammation and damage to the intestinal lining. Similarly, individuals with IBS often exhibit altered gut microbiota profiles, which can contribute to symptoms such as bloating, abdominal pain, and altered bowel habits. Furthermore, SCFAs have been shown to promote the production of mucus, enhance gut barrier integrity, and support the growth of beneficial bacteria, all of which are critical for maintaining gastrointestinal health. They also interact with host cells through specific receptors, influencing various metabolic and immune pathways.

### 3.4 Metabolism and weight management

The gut microbiota plays a crucial role in metabolism, influencing the body’s ability to extract and store energy from dietary intake. Variations in microbial composition can significantly affect weight gain, fat retention, and susceptibility to metabolic disorders, including obesity and type 2 diabetes (Geng et al., 2022). In individuals with obesity and related metabolic diseases, the composition and functionality of the gut microbiome exhibit distinct alterations. Mechanistic studies have shown that the gut microbiota impacts the energy balance equation in two primary ways: it can influence how energy is extracted from food and modulate host genes responsible for regulating energy storage and expenditure. A well-balanced microbiome is associated with healthy metabolic processes and effective weight management (Valdes et al., 2018). Recently, the concept of the gut-brain axis has gained attention, highlighting the intricate relationship between gut microbiota and mental health. This network encompasses not only anatomical pathways but also humoral, endocrine, metabolic, and immunological communication routes (Singh A. et al., 2024). The brain can regulate intestinal functions through several mechanisms, including the autonomic nervous system, the hypothalamic-pituitary-adrenal (HPA) axis, and intrinsic nerves within the gastrointestinal tract (Appleton, 2018). The gut microbiota influences mood and cognitive function by affecting inflammation, modulating stress responses, and synthesizing neurotransmitters. Disruptions in the gut microbiome have been linked to a variety of mental health issues, including mood disorders, anxiety, and depression (Clapp et al., 2017). This bidirectional communication illustrates the significant impact that gut health can have on brain function and overall wellbeing, underscoring the importance of maintaining a balanced and diverse gut microbiota for both metabolic health and mental health.

## 4 Synthetic biology approaches in microbiota engineering

Synthetic biology offers innovative tools and methodologies for engineering the microbiota, paving the way for new therapeutic and diagnostic applications. This interdisciplinary field allows researchers to manipulate microbial systems for a variety of beneficial purposes, including metabolic engineering, genetic modification, personalized medicine, and biosensing (Lu et al., 2023).

### 4.1 Metabolic engineering and biosynthetic pathway construction

One of the primary applications of synthetic biology in microbiota engineering is the development of engineered microorganisms capable of synthesizing valuable metabolites that are not produced by their wild-type counterparts. This approach has extensive applications in the production of chemicals, biofuels, food additives, and pharmaceuticals. By constructing synthetic biosynthetic pathways, researchers can enable bacteria to produce essential compounds such as vitamins, hormones, and anti-inflammatory agents. This strategy is particularly promising for developing novel probiotics with enhanced health benefits (Nielsen and Keasling, 2016).

### 4.2 Microbial genetic engineering and gene editing

Advancements in gene editing technologies, such as CRISPR-Cas9, have revolutionized the ability to precisely modify the genomes of bacteria. This capability allows for the targeted addition, removal, or alteration of specific genes to enhance desirable traits or mitigate adverse effects (Arroyo-Olarte et al., 2021). For example, genetically modified probiotics can be engineered to produce therapeutic compounds or enzymes that facilitate the breakdown of harmful toxins, thereby improving gut health and overall wellbeing (Zhou et al., 2020).

### 4.3 Personalized probiotics through synthetic biology

Synthetic biology techniques enable the development of probiotic strains tailored to individual patient needs. By customizing these probiotics to specifically address dysbiosis or deficiencies within the gut microbiota, researchers can offer more personalized and effective therapeutic options. This individualized approach enhances the potential for targeted interventions that align with each patient’s unique microbiome profile (Bober et al., 2018; Mugwanda et al., 2023). Personalized probiotics developed through synthetic biology represent a groundbreaking approach to enhancing human health by tailoring microbial therapies to individual microbiome profiles. By leveraging advanced genetic engineering techniques, these probiotics can be customized to support specific health needs, optimize gut function, and restore microbial balance (Yadav et al., 2022b).

### 4.4 Engineered biosensors and feedback systems

Engineered microbes can also function as sophisticated biosensors, capable of detecting specific environmental chemicals or conditions. These biosensors serve as advanced measurement tools, offering the ability to identify clinical pathogens such as bacteria and viruses with high accuracy. Modified microorganisms can generate observable signals, such as fluorescence or color changes, in response to environmental stimuli. This capability enables real-time monitoring of infections or gut health status, facilitating timely interventions (Castillo-Henríquez et al., 2020; Saifi et al., 2022).

### 4.5 Targeted drug delivery systems

**FIGURE 3:**
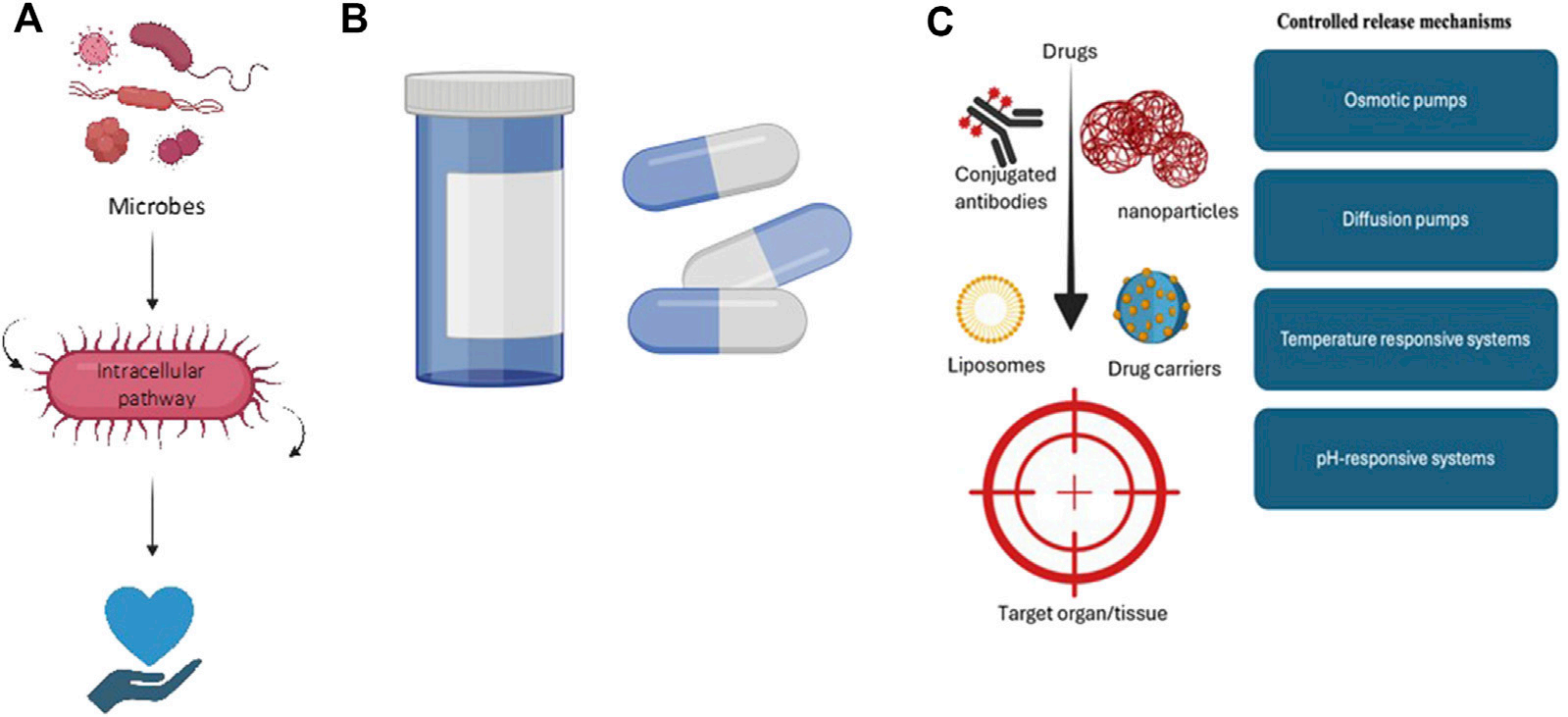
Various drug delivery methods, including **(A)** microbes as drug delivery vehicles, which improve treatment efficacy; **(B)** targeted delivery of therapeutics, which directs drugs to specific sites for increased effectiveness and **(C)** controlled release mechanisms, which allow for the sustained and gradual release of therapeutics.

Synthetic biology further allows for the design of microorganisms that can deliver therapeutic compounds to precise locations within the body, such as the gastrointestinal tract, demonstrated in Figure 3. These engineered bacteria can be programmed to release their therapeutic cargo in response to specific environmental triggers or conditions, thereby enhancing the accuracy and efficacy of treatments. This targeted delivery approach has the potential to minimize side effects and maximize therapeutic outcomes, representing a significant advancement in the field of microbial therapeutics.

## 5 Applications of synthetic biology in microbiota therapeutics

Synthetic biology is transforming microbiota therapeutics by enabling the design of engineered microbes that can precisely interact with the host microbiome. Unlike traditional probiotics, these engineered organisms are tailored to sense, respond to, and modulate specific conditions within the gut. This approach opens new avenues for treating diseases linked to microbiome imbalances, offering targeted interventions that traditional therapies cannot achieve.

### 5.1 Disease treatment and prevention

Synthetic biology is revolutionizing the treatment and prevention of various diseases through innovative approaches that harness the capabilities of engineered microbes. One of the most promising areas is the use of engineered probiotics to address gastrointestinal disorders. These probiotics are specifically modified strains of bacteria that can produce therapeutic compounds, enhance gut functionality, or deliver anti-inflammatory effects. For instance, genetically engineered probiotics may synthesize therapeutic peptides or cytokines that can alleviate symptoms of conditions like irritable bowel syndrome (IBS) and inflammatory bowel disease (IBD). By tailoring these probiotics to target specific pathological conditions, researchers aim to enhance their efficacy while leveraging the natural health benefits of beneficial microbes. Studies have shown that modified probiotics can effectively alter the gut microbiota composition, restoring balance and improving patient outcomes.

Synthetic microbiota and engineered probiotics offer encouraging treatment options for autoimmune, metabolic, and gastrointestinal conditions. Probiotics designed to generate therapeutic peptides, or anti-inflammatory cytokines have demonstrated effectiveness in reducing symptoms of gastrointestinal disorders such as IBD and IBS by reestablishing the balance of the gut microbiota (Ma et al., 2022; Pesce et al., 2022; Romero-Luna et al., 2022). For instance, mixed probiotics (NS) continuously raised the levels of beneficial bacteria, but *Lactobacillus* buchneri (SU) had varying impacts on them (Uchiyama-Tanaka, 2014). Additionally, a study conducted on IBS patients showed that fiber-enriched probiotic milk reduced bloating and discomfort and dramatically increased the frequency of bowel movements (Choi et al., 2011). By improving bile acid metabolism and SCFA generation, synthetic microbiota may help treat metabolic diseases including diabetes and obesity. This could enhance glucose metabolism and decrease the buildup of fat (Scheithauer et al., 2020; Wu et al., 2021). Higher risks of obesity, insulin resistance, and inflammation are linked to decreased gut microbial diversity (Crudele et al., 2023). Engineered probiotics that alter immune responses have the potential to improve autoimmune disorders like rheumatoid arthritis and multiple sclerosis (Wang et al., 2024). Probiotics are generally well-tolerated and do not significantly increase side effects, according to a study of 80 RCTs that demonstrated the efficacy of gut microbiota-based therapy in lowering symptoms in autoimmune illnesses (Zeng et al., 2024).

### 5.2 Drug delivery systems

Drug delivery systems play a pivotal role in enhancing the efficacy and safety of therapeutic interventions. These systems employ various strategies to optimize drug administration, targeting specific areas within the body while minimizing side effects (Coelho et al., 2010). Modern approaches in drug delivery encompass controlled-release formulations that dispense active ingredients at predetermined rates, alongside targeted delivery systems that direct therapeutic effects to particular tissues or cells (Khafoor et al., 2023). For instance, liposomal and nanoparticle-based systems (Yadav et al., 2023) have gained traction for their ability to encapsulate drugs, protecting them from degradation and improving absorption by target cells (Ezike et al., 2023). Moreover, advanced drug delivery technologies feature smart devices (Yadav et al., 2020a) capable of responding to physiological changes, such as alterations in pH or temperature, facilitating the release of drugs under specific conditions. These innovations not only enhance therapeutic outcomes but also reduce the frequency of administration and improve patient compliance (Nieuwlaat et al., 2014).

Microorganisms’ innate capacity to locate within certain tissues and react to environmental cues enhances therapeutic accuracy and opens up new avenues for drug administration. For instance, medicinal chemicals can be transported directly to target locations, such as the gastrointestinal tract, by engineered bacteria and yeast, enabling localized treatment and minimizing systemic side effects (Shende and Basarkar, 2019; Santos-Beneit, 2024). Furthermore, certain bacteria can be made to release medications in response to particular stimuli, such as temperature or pH, allowing for precise and regulated administration (Romero-Luna et al., 2022; Ma et al., 2022). By attaching to disease-specific markers and releasing medications in response to environmental changes, targeted delivery technologies like nanoparticles, liposomes, and antibody-conjugated carriers improve results and reduce off-target effects (Tewabe et al., 2021; Elumalai et al., 2024). Additionally, by delivering medications at steady rates over time, controlled release mechanisms—from matrix systems and reservoir membranes to osmotic pumps and responsive microencapsulation—improve safety and efficacy, especially in complex diseases like cancer (Adepu and Ramakrishna, 2021; Almoshari, 2022). When combined, these strategies offer a significant improvement in the efficacy and dispersion of treatments.

### 5.3 Diagnostic tools

Diagnostic tools in synthetic biology aim to improve the precision and effectiveness of engineered biological systems by facilitating the detection, monitoring, and analysis of synthetic constructs within biological contexts. These innovative technologies play a crucial role in validating synthetic biology applications, ensuring safety standards, and enhancing overall performance (Singh et al., 2019). By providing real-time insights and reliable data, these diagnostic tools are essential for advancing research and clinical applications in the field.

The diagnosis of gastrointestinal disorders and gut health monitoring are being revolutionized by biosensors and non-invasive diagnostic methods. In order to identify inflammation, dysbiosis, or the presence of pathogens, biosensors tailored to gut health use wearable technology or engineered microbes to detect biomarkers or environmental changes in real-time (Singh, Bansal and Pandey, 2019; Tanniche and Behkam, 2023). For example, ingestible biosensors can provide real-time insights by directly measuring gastrointestinal parameters like temperature or pH (De la Paz et al., 2022). By recognizing microbial DNA/RNA, advanced biosensors can identify pathogens, aid in illness diagnosis, and track microbial balance (Gradisteanu-Pircalabioru et al., 2024; Jeon et al., 2022). Additionally, non-invasive methods like MRI, ultrasound, and stool and breath studies enable the evaluation of gastrointestinal health without requiring invasive treatments. While stool and breath tests provide indicators for diseases including colorectal cancer, SIBO, and infections like *H. pylori*, ultrasound and MRI offer detailed views of internal organs that can diagnose inflammation and malignancies (Khannous-Lleiffe et al., 2022; Pham and Beauchamp, 2021). When used in tandem, these techniques provide useful, real-time data that makes it possible to control gastrointestinal health precisely and with less invasiveness.

### 5.4 Personalized medicine

Personalized medicine represents a groundbreaking approach to healthcare that customizes medical treatments and interventions based on the unique characteristics of each patient. This paradigm shift leverages information about an individual’s environment, genetics, and lifestyle to optimize treatment outcomes, enhance efficacy, and minimize side effects (Akhondzadeh, 2014).

Personalizing microbiota-based therapies, genetic, and metabolic profiling allows for highly tailored approaches to individual health needs. By analyzing a patient’s unique gut microbiome through advanced sequencing, healthcare providers can select specific probiotics or prebiotics to address microbial imbalances, improving treatment outcomes and minimizing side effects (Meher et al., 2024; Ji et al., 2023). Genetic profiling reveals DNA variations and mutations linked to disease susceptibility and drug response, enabling targeted therapies and optimizing medication choices (Malone et al., 2020; Hernandez and Blazer, 2006). Meanwhile, metabolic profiling examines metabolites in bodily fluids to identify metabolic imbalances, offering insights into conditions like diabetes and cardiovascular disease. This allows for personalized dietary and lifestyle interventions (Qiu et al., 2023). Furthermore, customized probiotics designed around individual gut microbiome composition and health factors provide a more precise alternative to traditional one-size-fits-all probiotics, enhancing digestion and overall wellbeing (Cunningham et al., 2021). Collectively, these personalized approaches mark a shift towards more effective, patient-specific healthcare solutions that integrate genetic, metabolic, and microbiome data.

## 6 Synthetic biology in the gut microbiome

Synthetic biology is revolutionizing our understanding and manipulation of the gut microbiome, providing novel therapeutic tools to address various health conditions. This field involves designing and engineering microbial strains with specific functions to modulate the gut ecosystem, influence metabolic processes, and improve disease outcomes (Arnold et al., 2023; Yang et al., 2023). By creating genetically modified microorganisms that can integrate with or influence the existing microbiota, synthetic biology enables targeted interventions in the gut environment with unprecedented precision (Leggieri et al., 2021).

One significant application of synthetic biology in the gut microbiome is the development of engineered probiotics, which are genetically modified strains designed to produce therapeutic compounds directly within the gut (Mendes et al., 2017). For instance, these probiotics can be programmed to secrete anti-inflammatory cytokines or other beneficial molecules, offering potential treatments for conditions like inflammatory bowel disease (IBD) and irritable bowel syndrome (IBS) (McCarty and Ledesma-Amaro, 2019). Engineered microbes can also release antimicrobial peptides or consume harmful metabolites, selectively targeting pathogenic bacteria while preserving beneficial species, which helps maintain or restore microbial balance (Mugwanda et al., 2023). Another innovative approach involves creating synthetic microbial communities that mimic or enhance natural microbiota functions. These synthetic consortia are carefully designed microbial groups engineered to perform specific tasks, such as enhancing short-chain fatty acid (SCFA) production, improving immune responses, or regulating metabolic pathways. Such consortia can potentially support metabolic health by modulating glucose metabolism and reducing inflammation, offering new strategies for managing obesity and type 2 diabetes (van Leeuwen et al., 2023).

Synthetic biology also enables real-time gut health monitoring through biosensors integrated into engineered microbes. These biosensor strains are designed to detect and respond to changes in the gut environment by emitting detectable signals in the presence of specific biomarkers, such as pH shifts or the presence of certain metabolites. These biosensors can be ingested and provide valuable information about gut health, helping clinicians monitor conditions like dysbiosis or infections without invasive testing (Ngashangva and Chattopadhyay, 2023; Sánchez-Tirado et al., 2023).

## 7 Challenges and future perspectives

While synthetic biology in the gut microbiome holds substantial promise, challenges remain. Ensuring the safety and stability of engineered organisms within the human body, preventing unintended interactions with native microbiota, and addressing regulatory concerns are key areas of ongoing research. Nonetheless, as technology advances, synthetic biology has the potential to transform gut microbiome-based therapies, allowing for highly customized, responsive treatments that address the complexities of individual gut health. A key challenge in synthetic biology is maintaining the genetic stability and control of engineered microorganisms, which can lead to unintended modifications that jeopardize project outcomes (Son et al., 2021). Effective management of gene expression and synthetic pathways is crucial, alongside addressing safety concerns regarding the environmental release of genetically modified organisms (Mahdizade Ari et al., 2024).

Looking ahead, advancements in synthetic biology are expected to enhance microbial engineering. Regulatory frameworks will need to adapt to address the unique challenges posed by engineered microbiomes, ensuring safety and efficacy through clear protocols for monitoring and containment (Yan et al., 2023; Mao et al., 2021; Kalidasan and Theva, 2021). Innovative techniques, such as CRISPR-Cas9, will allow for more precise development of synthetic microorganisms, potentially addressing global challenges like energy production and environmental degradation (Thurtle-Schmidt and Lo, 2018). Furthermore, the integration of systems biology will improve the resilience of synthetic pathways, while enhanced biosafety features, such as built-in kill switches, will strengthen containment measures. The rise of personalized applications, including tailored probiotics, will also be a significant focus area (Rottinghaus et al., 2022). Collaboration and open-source platforms will further accelerate advancements in the field (Koelmel et al., 2016).

## 8 Conclusion

In conclusion, synthetic biology stands at the forefront of innovation, harnessing principles from various disciplines to reshape healthcare, agriculture, industry, and environmental management. While it holds the promise of transformative solutions, the field must navigate ethical, regulatory, and technical hurdles. Progress in microbiota therapeutics exemplifies this potential, with engineered bacteria creating customized probiotics and advanced drug delivery systems that enhance treatment precision and efficacy. As personalized medicine continues to evolve, tailoring interventions to individual needs, the future of synthetic biology is poised to achieve greater accuracy and safety in addressing complex health challenges, driven by ongoing advancements and global collaboration.
